# A Multicountry Comparison of Three Coverage Evaluation Survey Sampling Methodologies for Neglected Tropical Diseases

**DOI:** 10.4269/ajtmh.19-0946

**Published:** 2020-08-24

**Authors:** Katherine Gass, Michael Deming, Roland Bougma, Franck Drabo, Edridah Muheki Tukahebwa, Square Mkwanda, Reina Teresa Velasquez, Rosa Elena Mejia, Pamela Sabina Mbabazi

**Affiliations:** 1 Neglected Tropical Disease Support Center, Task Force for Global Health, Decatur, Georgia;; 2 Children Without Worms, Task Force for Global Health, Decatur, Georgia;; 3 Direction de la Protection de la Santé de la Population, Ministère de la Santé, Ouagadougou, Burkina Faso;; 4 Vector Control Division, Ministry of Health, Kampala, Uganda;; 5 Lymphatic Filariasis Control Program, Ministry of Health and Population, Lilongwe, Malawi;; 6 Vigilancia de Zoonosis, Secretaria de Salud de Honduras, Tegucigalpa, Honduras;; 7 Organización Panamericana de Salud, Tegucigalpa, Honduras;; 8 Department of Control of Neglected Tropical Diseases, World Health Organization, Geneva, Switzerland

## Abstract

Coverage evaluation surveys (CESs) are an important complement to routinely reported drug coverage estimates following mass drug administration for neglected tropical diseases (NTDs). Although the WHO recommends the routine use of CESs, they are rarely implemented. Reasons for this low uptake are multifaceted; one is uncertainty on the best sampling method. We conducted a multicountry study to compare the statistical characteristics, cost, time, and complexity of three commonly used CES sampling methods: the Expanded Program on Immunization’s (EPI’s) 30 × 7 cluster survey, a stratified design with systematic sampling within strata to enable lot quality assurance sampling (S-LQAS) decision rules, and probability sampling with segmentation (PSS). The three CES methods were used in Burkina Faso, Honduras, Malawi, and Uganda, and results were compared across the country sites. All three CES methods were found to be feasible. The S-LQAS approach took the least amount of time to complete and, consequently, was the least expensive; however, all three methods cost less than $5,000 per district. The PSS design resulted in an unbiased, equal-probability sample of the target populations. By contrast, the EPI approach had inherent bias related to the selection of households. Because of modifications needed to maintain feasibility, the S-LQAS method also resulted in a non-probability sample with less precision than the other two methods. Given the comparable cost and time of the three sampling methods and the statistical advantages of the PSS method, the PSS method was deemed to be the best for CESs in NTD programs.

## INTRODUCTION

The success of neglected tropical disease (NTD) programs working to control or eliminate disease depends on their ability to deliver preventative chemotherapy (PC) to populations at risk.^1^ The drugs are delivered on an annual, semiannual, or biennial basis to populations living in endemic areas through mass drug administration (MDA) campaigns. The metric for determining if MDA campaigns have met their target is drug coverage, defined as the number of individuals in the program area (typically a district) who have swallowed the drug, or combination of drugs, divided by either the total population (“epidemiologic coverage”) or the population that was eligible to receive the drugs (“program coverage”). We know from experience and models that achieving high drug coverage, meaning coverage that meets or exceeds a disease-specific threshold set by the WHO, is expected to lead to the achievement of control and elimination goals.^2–4^

Monitoring of PC coverage is typically based on reported coverage rates, which are calculated by aggregating drug distributors’ records of doses administered and dividing by a population estimate typically taken from census projections. Although reported coverage is an essential tool for program monitoring, it is prone to errors resulting from incorrect estimates of the target population, weak health information systems, and, at times, intentional inflation of the number of persons treated.^5^ A coverage evaluation survey (CES) is a valuable tool for evaluating program performance. Coverage evaluation surveys are population-based surveys designed to provide adequately precise estimates of treatment coverage that overcome many of the biases and errors that can undermine reported coverage. Whereas typically regarded as a tool for estimating treatment coverage, the benefits of and uses of CESs go beyond the estimation of treatment levels attained. Because participant recall in CESs has been shown to be generally accurate, particularly when surveys are conducted shortly after MDA, CESs can be used by country programs to assess the accuracy of the routine reporting system.^6^ Other potential uses for CESs include identifying reasons for noncompliance, detecting problems with the supply chain and distribution systems, measuring coverage in specific subpopulations of interest, and serving as a vehicle for estimating the value of other variables, such as those related to knowledge, attitudes, and perceptions regarding MDAs.

The WHO recommends the routine use of CESs as a means of monitoring MDA programs, but they are rarely implemented on a routine basis.^7,8^ Anecdotally, national NTD programs and implementing partners commonly mention as reasons for the infrequent use of CESs that they are expensive, resource intensive, and difficult to conduct well, and that the results arrive too late to impact the current MDA. When conducted, CESs have traditionally used a variation of the sampling design developed by the Expanded Program on Immunization (EPI) to measure immunization coverage.^9^ Indeed, the 2011 WHO manual on the epidemiological assessment of MDAs to eliminate lymphatic filariasis (LF) recommends a variation of the EPI approach for CESs.^7^ However, it has long been recognized that the original EPI cluster survey approach may lead to biased results because it does not rely on probability-based sampling.^10^ Whereas the EPI approach is fast and simple, survey statisticians have proposed a probability-based cluster sampling design in which primary sampling units (PSUs) are divided into segments as a way to reduce the workload, with the sample selected within a single segment. Another sampling alternative to the EPI approach that has been put forth is lot quality assurance sampling (LQAS), in which random or systematic sampling is used to determine if a threshold coverage level has been reached.^11^ Lot quality assurance sampling surveys are typically conducted in smaller strata (e.g., subdistricts or supervision areas), and then results from each stratum can be aggregated to calculate a coverage estimate for the entire survey area.^12,13^ The LQAS approach is touted for its small sample size, the ease of basing programmatic decisions on the threshold results, and the ability to gain programmatically useful information at the subdistrict level.

Until 2016, there was a lack of a single recommended sampling design for conducting CESs across the PC NTDs, which contributed to poor uptake of the surveys by country programs. As a result, the decision of whether to conduct a CES and the methodology used has been left to implementing partners and disease-specific focal points.

In 2013, the Task Force for Global Health convened a stakeholders’ meeting on CESs to better understand the reasons for their infrequent use. A key output of the meeting was the need to conduct a rigorous comparison of the programmatic feasibility of the most common sampling methodologies, with the goal of developing a single set of guidelines for conducting CESs for PC NTDs.

To address the stakeholder meeting recommendation, this multicountry study was designed to compare the cost, time, and complexity of CESs using the following sampling methods: the EPI approach, stratified survey using lot quality assurance sampling (S-LQAS) within strata, and probability sampling with segmentation (PSS). The three sampling methods were compared in Burkina Faso, Honduras, Malawi, and Uganda, always within the context of the ongoing NTD program. This article describes the sampling methods under comparison, interprets the resulting PC coverage estimates, compares the different feasibility metrics, and ultimately weighs the statistical properties and programmatic feasibility of each method to recommend the one best suited for NTD programs.

## METHODS

The EPI, LQAS, and PSS methods were each piloted at the district level (or district-level equivalent) in Burkina Faso, Honduras, Malawi, and Uganda (Table 1). The following is a brief description of the sampling methods and how they differed from each other.

**Table 1:** Summary of coverage evaluation survey characteristics by country and district

Country	District	Sampling methodology	Primary sampling unit	Disease(s) targeted	Drugs assessed	WHO target coverage threshold (%)	Survey population
Burkina Faso	Batie	PSS	Enumeration areas	LF	Albendazole and ivermectin	65	Everybody (all ages)
	Dano	S-LQAS	NA				
	Diebougou	EPI	Enumeration areas				
Honduras	Gracias	EPI	Village	Soil-transmitted helminthiasis	Albendazole	75	Children aged 24–59 months
	Lepaera	S-LQAS	NA				
	San Juan	PSS	Village				
Malawi	Balaka	S-LQAS	NA	LF	Albendazole and ivermectin	65	Everybody (all ages)
	Machinga	EPI	Village				
	Zomba	PSS	Village				
Uganda	Amuru	EPI, S-LQAS, and PSS	Village	LF, onchocerciasis, schistosomiasis, and trachoma	Albendazole, ivermectin, praziquantel, and azithromycin	65, 65, 75, and 80*	Everybody (all ages)

EPI = Expanded Program on Immunization; LF = lymphatic filariasis; NA = not applicable; PSS = probability sampling with segmentation; S-LQAS = stratified survey with lot quality assurance sampling.

* WHO target coverage thresholds for lymphatic filariasis, onchocerciasis, schistosomiasis, and trachoma, respectively.

### Key terms.

The PSU definition was consistent across all three sampling methods within each country and typically corresponded to the smallest administrative unit for which a population estimate could be obtained. Primary sampling units were typically villages, including localities and hamlets, or census enumeration areas (EAs). An EA is generally the area canvassed by a single census worker. The size of EAs in households at the time of the last census and EA maps are obtained from the country’s census office. Enumeration areas generally have fewer households and vary less in the number of households than other units used as PSUs, and have the desirable attributes for probability sampling described in the discussion section of this article. Because of these advantages, EAs are used as PSUs in Demographic and Health Surveys and in UNICEF’s Multiple-Indicator Cluster Surveys.^14,15^

The target population is defined as the population for which an estimate of PC coverage was desired. For CESs measuring MDA coverage for LF, onchocerciasis, or trachoma, the target population was everybody living in the targeted district, regardless of MDA eligibility. This aligns with the WHO identified targets for epidemiologic coverage (Supplemental Table 1).

### The EPI’s cluster sampling approach.

The EPI approach is a cluster sampling approach for calculating coverage that has been implemented thousands of times around the world.^9^ It is designed to be as quick and easy as possible while ensuring that data are collected throughout the sampling frame. This study followed the protocol described by the Global Program to Eliminate LF, which is based on WHO’s EPI sampling methodology.^7,9^ In brief, 30 clusters were selected from a list of all PSUs in the district using probability proportional to estimated size sampling (PPES) using total population as the measure of size. On arrival at each selected cluster, the survey team asked the local leaders to identify the center of the PSU. The starting household was chosen by picking a random direction from the center of the PSU by spinning a pen or bottle, enumerating all households in the chosen direction up to the outer border of the PSU, and then picking one at random to be the first selected house. Household sampling proceeded by selecting the nearest-neighbor household, defined as the household with a front door closest to the previously selected household. Within each selected household, all members of the target population were interviewed. This process continued until the target sample size for the cluster (the same for each cluster) was obtained. If the sample size was met before enrolling everyone in the final household, then enrollment continued until all household members were included.

### Stratified sample using lot quality assurance sampling within each stratum.

Lot quality assurance sampling is a random sampling method that was originally developed by the manufacturing industry and later applied to public health.^16,17^ Generally, a small simple random sample is taken from a population (or “lot”), and the results are used to classify the lot as “acceptable” or “unacceptable” relative to a given threshold. With the lots treated as strata, prevalence estimates and standard error can then be calculated for the lots as a whole.

Districts selected for the stratified sample using LQAS within each stratum (the S-LQAS approach) were first divided into five supervisory areas (SAs) that corresponded to subdistrict areas of MDA supervisory responsibility. These SAs were the survey strata. First, 19 villages or EAs were selected systematically from within each SA using PPES sampling with the number of households as the measure of size. It was possible for a large village or EA to be selected more than once. Within each selected village or EA, a single household was chosen; two households were chosen if the village or EA was selected twice. Whenever possible, household selection was performed by picking a random number between one and the total number of houses in the village or health system register; the selected household was the one corresponding with the random number. When a register was unavailable, the survey team asked the village leaders to make a list of all households and randomly pick one. If a register was unavailable and the village/EA was too large for the village leader to enumerate, then the team first divided the PSUs into segments of approximately equal size in households, randomly selected one, and then enumerated all households in the selected segment and randomly selected one.

To select a single respondent from the selected household, names of everyone living in the household, regardless of MDA eligibility, were listed on a piece of paper that was placed in a hat or bowl. One name was drawn, and that person was enrolled in the study. If the selected individual was not present, then another member of the household was asked to serve as a proxy respondent for the absent individual. In the event that a proxy was not present or if the selected household was empty, the nearest-neighbor household was visited and a single individual chosen for enrollment, as described earlier.

### Probability sampling with segmentation.

The PSS design produces an equal-probability sample of the target population and is derived from the “modified segment design” option described in the manuals for rounds 2–4 of UNICEF’s Multiple-Indicator Cluster Surveys and by Turner et al.^18,19^ First, the sampling team constructed a list of all PSUs in the survey area, each with its projected number of households. If PSU size in households was not already available, then it was calculated by dividing each PSU’s projected total population by the average household size. The estimated number of households in each PSU was then divided by 50 and rounded to the nearest integer to determine the number of 50-household segments to assign to each PSU. Next, 30 PSUs were selected from the list of all PSUs in the survey area by PPES sampling using the number of segments per PSU as its measure of size. On arrival at a selected PSU, the survey team worked with the local leaders to divide the PSU into a predetermined number of segments equal to the number of segments used for the PPES selection of the PSU. Segments within a PSU were delineated such that each had approximately the same number of households. One segment was selected at random, and a fixed proportion of households was selected systematically from among all households in the segment. The same proportion was used in all selected segments and was expected to produce the overall target sample size. All residents of the selected households were enrolled in the survey. If one or more residents of a selected household were not present at the time of the survey, then a family member was asked to serve as a proxy respondent and respond on their behalf. If no proxy was available, then the absent individual was excluded from the survey; there was no selection of make-up households because the sample size included inflation for nonresponse. The reason for defining PSU size as the number of 50 households it contains is based on field experience, indicating it would result in an efficient balance for NTD CESs between the time needed for segmentation (increased by segments with fewer households) and the time needed to enumerate and visit households within segments (increased by segments with more households). As shown by Equation (2), the PPS method results in an equal-probability sample regardless of whether a higher or lower number than 50 is used to define the segment size.

Within-segment sampling fraction of households (fixed across all segments):

f= target sample size 30×avg. HH size×50(1−r), (1)

where HH = household, *f =* sampling fraction of households, and *r* = anticipated rate of nonresponse.

Probability of selection for a target population member living in the survey area:

P(any target population memberl living in PSUi, segmentj, HHk)= 30 (# segments PSUi)sum of segments across all PSUs × 1(# segments PSUi) × f×  # target pop members in HHk# target pop members in HHk,= 30 f sum of segments across all PSUs, (2)

where PSU = primary sampling unit (i.e., village or EA) and pop = population.

### Sample size.

Whenever possible, the statistical properties and assumptions required to calculate the sample size for each method were held constant across the different sampling methods for greater comparability. The required sample size for both the EPI and PSS surveys was 1,537 (1,808 after inflation for 15% nonresponse) and was calculated using the following formula, where design effect (DEFF) is the anticipated DEFF, $Z_{\text{α}/2}^{2}$ is the critical value of the normal distribution at α/2, *P* is the expected proportion of coverage (the most conservative value of *P* = 0.5 was used), δ is the desired level of precision (with the exception of Honduras, all sites used ±5% precision for both the EPI and PSS surveys), and *r* is the anticipated nonresponse rate (Table 1).

$$n=\frac{\;\left(\text{DEFF}\right)\left(Z_{\text{α}/2}^{2}\right)\left(P\right)\left(1-P\right)}{\delta^{2}\left(1-r\right)}.$$ (3)

For S-LQAS, the sample size was set at the SA level, and a decision rule was specified for classifying coverage within the SA. The decision rule depends on two types of error: wrongly classifying coverage as below a threshold when it is at or above the threshold (type I) and wrongly classifying coverage as at or above a threshold when it is below the threshold (type II). A sample size of *n* = 19 was deemed appropriate for classifying coverage at thresholds of 65%, 75%, and 80% (the WHO target thresholds for the PC NTDs), while keeping the risk of both alpha and beta errors less than 10%. Results were aggregated across the SAs to calculate a weighted coverage estimate for the entire district, taking account of the stratified survey design. To achieve a coverage estimate with comparable precision (±5%) and confidence level (95%) to the EPI and PSS designs would have required visiting 384 separate villages/EAs per district, which was deemed infeasible. Instead, it was decided that (±10%) precision, at the same 95% CI, was acceptable and resulted in a feasible sample size of 96. This was reduced to 95 so that it resulted in an integer when divided by five (the number of SAs). Therefore, 19 people were selected in each SA (Table 2).

**Table 2:** Statistical parameters used for sample size calculations across the three sampling methods

	Expanded Program on Immunization’s cluster survey	Stratified survey with lot quality assurance sampling within strata	Probability sampling with segmentation
Expected coverage (*P*) (%)	50	50	50
Desired precision (δ) (%)	5	10	5
Confidence level (*Z*) (%)	95	95	95
Anticipated nonresponse (*r*) (%)	15	NA	15
Anticipated design effect	4	NA	4
Target sample size* (*n*)	1,537	95†	1,537
Stratum	Single stratum consisting of entire target population	Target population divided into five strata (SAs)	Single stratum consisting of entire target population
Selection of PSUs	30 PSUs selected with probability proportional to the estimated size in total population	19 EAs/villages selected systematically from within each stratum with probability proportional to estimated size in households	30 PSUs selected with probability proportional to estimated size in segments
Selection of secondary sampling units	Random walk, and enrolling nearest-neighbor households until PSU quota for each PSU is reached	One household selected randomly within each selected EA/village	PSUs selected for the survey sample segmented, and then one segment was selected at random. Fixed sampling interval used to systematically select households within selected segments
Selection of individuals within selected households	All eligible persons	A single person selected randomly from among all household members	All eligible persons

EA = enumeration area; NA = not applicable; PSU = primary sampling unit; SA = supervisory area.

* Target sample size does not reflect the inflation for nonresponse.

† Stratified survey with lot quality assurance sampling information is for the district-level results from the stratified and weighted analyses of the five strata SAs.

To assist the survey teams with the sample size calculations and site selection, an Excel-based tool was developed that includes separate calculation sheets for each methodology (accessible at: https://osf.io/j9wgr/).

### Burkina Faso.

The CESs took place in Burkina Faso in February–March 2015 and were used to evaluate the coverage of a recent LF MDA (ivermectin and albendazole) that had taken place 6 months before the assessment. The Ministry of Health formed three survey teams, each of which was trained on one of the sampling methods and assigned to a separate district. The EPI survey was implemented in Diebougou district, the S-LQAS survey in Dano district, and the PSS survey in Batie district. As per the study protocol, districts were selected to be as similar as possible with regard to population density, area size, and endemicity of NTDs. In addition to the standard CES questionnaire, the national NTD programs added questions to assess the population’s knowledge, attitudes, and perceptions related to the LF MDA.

### Honduras.

In Honduras, the CESs were conducted in June–July 2015 and were used to measure the deworming coverage (albendazole) among preschool-aged children (PSAC; defined here as 24–59 months) 1–2 months after the MDA. Five survey teams were formed: two teams jointly implemented the EPI approach in Gracias district, two teams jointly implemented the PSS approach in San Juan district, and one team implemented the S-LQAS in Lepaera district. These districts were selected based on the program need, safety for study teams, and similarity of demographic and epidemiologic characteristics. The Honduran program determined that ±10% precision was sufficient for all three sampling methods, resulting in a smaller sample size for the EPI and PSS surveys than the other countries. Furthermore, because the target population was only 24–59 months, Equation (1) was modified for the EPI and PSS surveys in Honduras, replacing the average household size with the average number of children aged 24–59 months per household. For the S-LQAS survey, if the selected household did not have a child in the target age range, then a replacement household was randomly chosen. The CESs were conducted as an integrated activity with the EPI to measure coverage of measles vaccine.

### Malawi.

The Malawi CESs were conducted in February of 2014 and were used to evaluate the coverage of an LF MDA (ivermectin and albendazole) that had taken place 6 months prior. The districts of Machinga, Balaka, and Zomba were selected for the study, based on the needs of the program and the demographic and epidemiologic similarities of the districts. Three district-level teams were identified by the MOH and each trained on one of the sampling methods: the EPI method in Machinga district, the S-LQAS method in Balaka district, and the PSS method in Zomba district.

### Uganda.

In Uganda, the CESs were conducted in October–November 2014. To accommodate the National Program’s request to evaluate multiple drug packages, integrated CESs were conducted to measure the coverage for LF and onchocerciasis (ivermectin and albendazole), distributed 6 months prior, as well as coverage for schistosomiasis and trachoma (praziquantel and azithromycin, respectively), distributed 4 months prior. Survey team members were trained on approaches to help improve participants’ ability to recall. Techniques included letting participants hold examples of each pill while describing the unique aspects of each MDA (e.g., “do you remember standing against the tall dosing pole?”) and reminding participants which diseases each drug treats. Because Amuru district is considered a high-risk area for schistosomiasis, both children and adults were eligible to receive praziquantel. Consequently, everybody living in the district was considered part of the target population for the integrated CES. To obtain a more meaningful comparison of the relative feasibility of the sampling methods, the Ministry of Health decided to conduct all three sampling methods in the Amuru district. Three survey teams were formed at the national level and trained on all three methods. The teams then worked together to conduct three independent surveys in Amuru district, starting with the PSS sampling method, followed by the S-LQAS method and then the EPI method. As the selection of PSUs was performed independently for each sampling method, in some instances, the same PSU was included in more than one coverage survey and received multiple visits from the survey teams.

### Team composition.

Each survey team comprised one driver and two surveyors. One surveyor served as the record-keeper, in charge of recording the time of arrival and the survey responses in the handheld electronic device and one interviewer in charge of interviewing the respondents and showing examples of medication offered during the MDA. On arrival in a selected PSU, the survey team introduced themselves to the local leader and sought his or her permission to implement the CES. Once permission was granted, the survey team identified a local guide (generally a health worker or village leader) to help navigate the PSU and introduce the survey team at the selected households.

### Survey questionnaire.

At each selected household, the survey team members introduced themselves to the head of household and sought verbal consent for each family member to participate in the survey. All members of the target population were considered eligible to participate in the survey. To increase participant recall, individuals were shown examples of the drug(s) that were offered during the MDA before being asked to respond to the survey questions. Caregivers were asked to respond on behalf of children aged < 10 years. If a respondent was missing but expected to return the same day, then the survey team returned later the same day. If the respondent was still missing at the second visit or missing and not expected to return at the time of the initial visit, then another adult in the household was invited to answer on behalf of the missing respondent. Such answers were noted as proxy responses in the database. In the event that no one was present at a selected household, the household was skipped, and the survey team proceeded to the next selected household.

### Feasibility.

To measure the relative feasibility of each sampling methodology, the survey teams were asked to record the time of arrival and departure at each PSU. This information was validated against the automatic time and date stamps recorded by the cell phones at the initiation and completion of each survey record. At the end of the survey, the teams calculated the number of days it took to complete the survey fieldwork, the mean time (in minutes) to finish sampling within a PSU, and the cost of the survey. In addition, at the end of the CES, each team member was asked to complete a qualitative questionnaire to assess their feelings regarding the complexity, challenges, and overall impression of the sampling methodology to which they were assigned.

### Data collection.

All data were collected using the LINKS system (Task Force for Global Health, Decatur, GA) on Android-based cell phones.^20^

### Ethics.

Ethical clearance from the local institutional review boards was sought in advance of each study. In Burkina Faso, ethical clearance was granted from the Ethical Committee for Health Research in the Ministry of Health. In Honduras, ethical clearance was provided by the Pan American Health Organization’s Ethical Review Committee. In both Malawi and Uganda, the Ministries of Health considered the CES to be part of routine public health program practice and each sent a formal letter indicating that ethical approval was not necessary for the study. Permission to conduct the survey was obtained from community leaders on arrival in each PSU, and all participants gave verbal consent before participating in the survey.

### Analysis.

Drug coverage was calculated as the number of people who reported having swallowed the drug(s) divided by everyone enumerated in the survey area (“epidemiologic coverage”). The one exception was for coverage of albendazole in Honduras, where the denominator was the number of PSAC in the survey area. For the S-LQAS survey, the SA coverage results were weighted according to the projected total population of each SA to arrive at the district-wide estimate. Confidence limits for the EPI and PSS methods were calculated using the paired selection model, described by Kish, which accounts for the implicit stratification that occurs when PSUs are selected from a geographically ordered sampling frame and for variance inflation due to the correlation of responses within each PSU.^21^ Confidence limits for the S-LQAS survey were calculated by treating the survey as a stratified sample. Data cleaning and basic analyses were performed in SAS v9.4 (SAS Institute, Cary, NC), whereas the complex survey analysis was conducted in R version 3.3.1 (R Core Team, Vienna Austria) using the “survey” package.^22,23^

## RESULTS

A total of 11,094 people were interviewed for the 12 independent coverage surveys, conducted across the four countries (Table 3). The surveyed coverage estimates varied widely by country, ranging from 13% to 86%. The DEFF for survey coverage ranged from 0.9 to 22.68, with a median value of 2.14.

**Table 3:** Coverage evaluation survey results by country and sampling methodology

Country	District	Method	Drug(s)	*N*	Number who swallowed drug(s)*	Surveyed coverage (%)	Two-sided 95% CI	Design effect	Coverage above WHO threshold?	Reported coverage (%)	Validation of reported coverage	Lower 1-sided 95% CI
Burkina	Diebougou	EPI	ALB + IVM	1,821	1,464	80	(78, 83)	1.68	Yes	84	No	78
	Dano	S-LQAS	ALB + IVM	95	73	74	(64, 85)	1.49	Yes	83	Yes	66
	Batie	PSS	ALB + IVM	1,783	1,477	83	(81, 85)	1.3	Yes	81	Yes	81
Honduras	Gracias	EPI	ALB	473	249	53	(45, 60)	2.28	No	89	No	46
	Lepaera	S-LQAS	ALB	92	42	43	(33, 54)	1.08	No	42	Yes	35
	San Juan	PSS	ALB	444	258	58	(52, 64)	1.41	No	76	No	53
Malawi	Machinga	EPI	ALB + IVM	1,851	1,359	73	(70, 77)	2.48	Yes	89	No	70
	Balaka	S-LQAS	ALB + IVM	94	79	86	(79, 93)	0.97	Yes	87	Yes	80
	Zomba	PSS	ALB + IVM	1,581	1,212	77	(73, 80)	2.14	Yes	83	No	74
Uganda	Amuru	EPI	ALB	1,637	648	40	(27, 52)	22.49	No	79	No	29
			IVM	1,641	806	49	(39, 60)	16.23	No	78	No	41
			PZQ	1,606	339	21	(13, 30)	15.56	No	75	No	6
			ZITH	1,633	304	19	(10, 27)	18.71	No	72	No	11
		S-LQAS	ALB	85	38	44	(33, 56)	1.18	No	79	No	35
			IVM	84	40	4	(35, 59)	1.13	No	78	No	37
			PZQ	82	14	13	(7, 20)	0.8	No	75	No	8
			ZITH	82	18	21	(11, 31)	1.18	No	72	No	13
		PSS	ALB	1,205	568	47	(36, 58)	12.36	No	79	No	38
			IVM	1,219	635	52	(43, 61)	9.04	No	78	No	44
			PZQ	1,198	316	26	(15, 38)	18.58	No	75	No	17
			ZITH	1,201	269	22	(10, 35)	22.68	No	72	No	12

ALB = albendazole; EPI = Expanded Program on Immunization; IVM = ivermectin; PSS = probability sampling with segmentation; PZQ = praziquantel; S-LQAS = stratified survey with lot quality assurance sampling; ZITH = azithromycin.

* Participants who responded “not sure” to the question of whether they swallowed the drug (*n* = 1,018) were removed from the analysis (both numerator and denominator); this accounted for 4.5% of total respondents.

In Burkina Faso, the results from all three surveys indicate that MDA coverage exceeded the WHO target threshold for LF (surveyed coverage ≥ 65%). In Dano and Batie districts, the reported coverage fell within the 95% CI for the surveyed coverage, serving as a validation of the reported coverage and an indication that the reporting system is working well. In Diebougou district, the reported coverage narrowly exceeded the upper confidence limit of the coverage estimate by survey (Table 3).

The surveyed coverage estimates from Honduras indicate that none of the three district sites achieved the WHO target threshold of ≥ 75% coverage for soil-transmitted helminthiasis. In Lepaera district, the reported coverage (42%) was included within the CI around the coverage estimate by survey (33% and 54%), serving as validation of the reported coverage, whereas in Gracias and San Juan districts, the reported coverages of 89% and 76%, respectively, far exceeded the upper confidence limit around the coverage estimate by survey (Table 3).

In Malawi, the coverage estimates by survey for all three districts exceeded the WHO target threshold of ≥ 65% coverage for LF; however, only in Balaka district was the reported coverage also validated by the survey coverage results. In Machinga and Zomba districts, the reported coverage (89% and 83%, respectively) exceeded the upper confidence limit around the coverage estimate by survey (Table 3).

Finally, in Uganda, the three sampling methods were used independently in the same district and separate estimates of coverage were obtained for each drug offered. All three sampling methods found that coverage for praziquantel and azithromycin was the lowest of the four drugs, and far below the MDA target thresholds of 75% and 80% for schistosomiasis and trachoma, respectively. Although the surveyed coverage estimates for albendazole and ivermectin were higher, they too fell short of the 65% target coverage by all three sampling methods. The reported coverage was universally high, relative to the surveyed coverage, for all four drugs; none of the three coverage sampling methods resulted in estimates that validated the reported coverage (Table 3).

In addition to the 2-sided 95% CIs around the survey coverage estimates, Table 3 also contains the lower 1-sided 95% CIs for each of the survey coverage estimates. It is important to note that the former is most useful when comparing the survey coverage with the reported coverage to see if the two figures are reasonably close (e.g., Is the reported coverage within or close to the 95% CI around the surveyed coverage?). The lower 1-sided CI is preferred for comparing with the WHO target threshold. If the lower 1-sided CI is equal to or greater than the target threshold, then one can be reasonably confident that the coverage meets or exceeds the recommended target.

The results from the feasibility calculations are shown in Table 4. The S-LQAS approach was the fastest to complete in every country except Burkina Faso, requiring more than 2 weeks (15 days). On average, the EPI and PSS approaches took the same number of team days to complete (19); however, the PSS approach took about a half hour longer per site than the EPI approach. The cost for all three approaches was similar, ranging from $3,200 to $4,500, once training costs were factored in.

**Table 4:** Time and cost calculations for the three coverage sampling methods

	Expanded Program on Immunization’s cluster survey			Stratified survey with lot quality assurance sampling within strata			Probability sampling with segmentation		
Country	Days required for one team to complete the survey	Mean time to complete sampling in each PSU (minutes)	Cost (training + implementation) ($)	Days required for one team to complete the survey	Mean time to complete sampling in each PSU (minutes)	Cost (training + implementation) ($)	Days required for one team to complete the survey	Mean time to complete sampling in each PSU (minutes)	Cost (training + implementation) ($)
Burkina Faso	18	276	4,385	19	59	4,816	17	277	4,525
Honduras	22	128	1,867*	9	19	1,167*	18	164	1,520*
Malawi	14	–†	4,113	10	–†	3,247	16	–†	4,546
Uganda	23	180	4,040	21	29	3,835	26	231	4,535
Average	19	195	3,601	15	36	3,266	19	224	3,782

PSUs = primary sampling units.

* Does not include training costs (e.g., facility rental, refreshments, transport of attendees to training site, and photocopies).

† Data not available.

**Figure 1.**
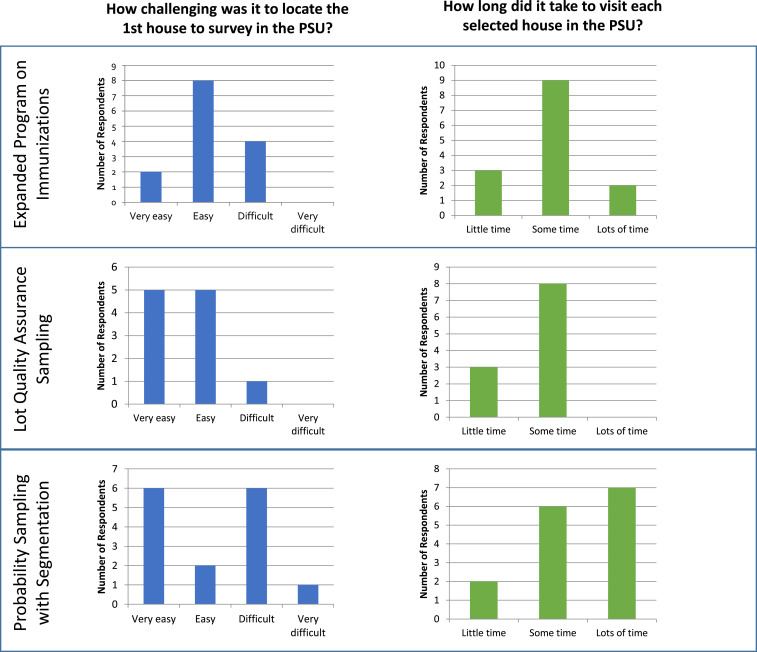
Survey team members’ responses to the qualitative questionnaire assessing the feasibility of each survey method; responses are summarized across all four countries. This figure appears in color at www.ajtmh.org.

Summary results from the qualitative questionnaire (Figure 1) suggest that survey team members’ perceptions varied widely within each method. The steps required to find the first house to survey within a given EA/village differ across the three sampling methods and are often time consuming. For the EPI approach, most respondents found it “easy” to find the first house. For the S-LQAS approach, most respondents found it “very easy” or “easy” to find the first house, whereas for the PSS approach, survey team members differed in whether it was “very easy” or “difficult.” The amount of perceived time it took to complete the sampling within a given PSU (stratum for S-LQAS) was considered to be the least for the S-LQAS approach, followed by more time for the EPI approach and the most perceived time for the PSS approach, although responses varied within each category.

## DISCUSSION

Coverage evaluation surveys are an important tool, used by public health programs to monitor the reach of an intervention. For NTD programs, CESs are essential for understanding the expected impact of MDAs, assessing the accuracy of the routine reporting system, and identifying ways to improve program performance. Because of the many sampling methods and adaptations that have been used for coverage surveys over the years, there have been several studies conducted to compare the coverage estimates and survey characteristics of different sampling methods for vaccination coverage^24–28^ and NTDs^29–31^; however, we are not aware of any studies that have also compared the feasibility of different coverage sampling methods in the context of NTD programs. This study was designed to compare the feasibility and statistical merits of three common coverage sampling methods (the EPI’s cluster survey, a stratified survey with LQAS within strata, and cluster sampling with segmentation) across a variety of country settings and disease platforms, with the aim of recommending a single CES sampling method for NTD programs to adopt moving forward.

All three CES methods were found to be feasible, in the sense that they were successfully conducted by national NTD programs using local staff. Overall, the S-LQAS approach was the fastest method to complete, requiring one team on an average of 14 days to complete the survey in the field; consequently, it was also the least expensive. The PSS approach required the most time to complete sampling within a PSU; however, overall, the EPI and PSS approaches took the same amount of time to complete the full survey (19 days). A closer look at the variation in time to completion suggests that the differences may have more to do with the characteristics of the districts themselves than the sampling methodology. For example, in districts with lower population density, the surveys took longer and team members had to walk further to find the next household, regardless of the method used. The Uganda study was designed to provide the best comparison of feasibility across the three sampling methods (the same teams conducted all three methods in the same district); however, it is hard to disentangle potential confounding due to the survey teams’ ability to adapt and improve over time. The teams reported that the work got easier and faster as they went on, with team members scoring the first approach (PSS) as the most difficult and the last approach (EPI) as the least difficult. For the field teams, the practical difference between the sampling methods relates mainly to the selection of the starting household within each PSU, the most time-consuming step. When asked to rank the difficulty of selecting the starting house, all three methods received scores ranging from “very easy” to “difficult,” with the PSS approach receiving the only “very difficult” score.

Although important, feasibility alone is not sufficient for identifying an optimal sampling method. The statistical qualities of the survey design merit careful consideration to ensure that the survey results are sufficiently accurate for program decision-making. The potential bias inherent in the EPI approach, whereby households have different but undefinable probabilities of selection, cannot be corrected for in the analysis. It is worth noting that because of this potential for bias, the EPI recently abandoned its classic 30 × 7 cluster methodology (the approach on which the “EPI” method described in this article is based) in favor of a more robust sampling method using segmentation.^32^

Stratified survey with lot quality assurance sampling can, in principle, result in an unbiased estimate of district coverage by combining results across the SAs, as long as every person in the survey sample has a known probability of selection. This could be achieved by recording the number of members in each selected household, randomly selecting one and then weighting that member’s response by the inverse of their selection probability. However, for the SA-level classifications to be valid, every individual must have an equal probability of selection. Selection probabilities would be equal if all members of a household were selected for the sample, as opposed to randomly selecting one person per household as was done in this study. Sampling 19 households, as opposed to 19 individuals, would constitute a “cluster LQAS” sampling design.^33^ Although this design may be worth considering for future surveys, it was deemed impractical in this instance because the need to account for clustering in the analysis would make it hard to interpret the LQAS decision rules. As a consequence of selecting only one individual per household, individuals living in larger households had a lower probability of selection than those living in households with fewer residents. These unequal selection probabilities become a bias concern if household size is associated with MDA coverage.

It is also important to point out that the sample size used in this study for the S-LQAS design resulted in significantly less precision than the other two methods (±10%, as opposed to ±5% for EPI and PSS); achieving comparable precision with the S-LQAS approach would require visiting 384 different PSUs. An important benefit of the S-LQAS design is the ability to classify coverage within an SA as above or below the target threshold within each survey area. Although insightful, because MDA decisions are often made on at a district level, this SA information did not change programmatic conclusions.

Of the three sampling methods compared here, only the PSS method results in a probability sample and is the favored approach for conducting CESs. In addition to the benefits of unbiased sampling, because the PSS results in an equal-probability sample, the point estimate can be interpreted directly, without the need for weighting or statistical adjustments, a nontrivial advantage for country teams that often have limited access to statistical software. Although this method requires survey teams to segment each selected PSU, when asked to rank the difficulty of this task, most team members found segmentation “easy” to perform. Segmentation became increasingly difficult for the survey teams in more populous PSUs, which require dividing the PSU into a greater number of segments. One way to avoid this challenge is to use EAs as PSUs instead of villages. Enumeration areas have several advantages over villages as PSUs: they are jointly exhaustive and mutually exclusive (every household falls in one and only one EA); they are relatively consistent in population size and generally have smaller populations than other units that have been used as PSUs; and they perform well in both urban and rural settings, and EA maps, often available now with GPS-defined borders, are typically available from the census office as well as spreadsheets with EA size in households at the time of the last census. Some country programs have reported difficulty in obtaining these documents from the census office; this may occur less frequently if the national EPI program, which has adopted a similar survey methodology, joins the NTD program in requesting them. Because the feasibility of the PSS approach is comparable to that of the other two methods considered here, its statistical advantages make it the preferred approach for conducting CESs.

Although the PSS approach may be the favored method for CESs, it is important to note that even with some sampling biases, all three sampling methods provided useful information back to the programs. Not only did the surveys provide information on whether target thresholds were being met and if the routine coverage reporting system was working well but because they were supplemented with additional questions regarding knowledge, attitudes and practices, they also provided more detailed information that enabled the programs to diagnose where implementation challenges lie. For example, in Burkina Faso, the team learned that most survey respondents heard about the MDA from a town crier, as opposed to radio spots or posters. In Malawi, the team learned that MDA refusals were very rare and that most instances of no treatment were due to individuals being away from home at the time of MDA. The results from Uganda enabled the program to identify a critical but previously undetected problem with the supply chain system that resulted in too few drugs being allocated to the district. The challenges with the supply chain also explain the exceedingly high DEFF observed in Uganda (Table 3), where coverage at the PSU level ranged dramatically from 0% to 100%. Finally, in Honduras, the survey results helped the national program to identify an issue of poor compliance due to unprogrammed deworming (i.e., many parents were deworming their children outside of the health campaign and consequently declining the deworming delivered through MDA). This information generated from each of the surveys demonstrates the benefit of conducting a CES, regardless of the survey methodology.

A challenge and potential limitation of any CES is the accuracy of self-reporting. There is a dearth of literature recall reliability for NTDs, whereas the literature for caregiver recall in childhood vaccination surveys suggests that the figures can vary quite widely.^34^ Recall reliability is of particular concern for integrated surveys, in which respondents are asked to differentially recall their ingestion of two or more drugs, or two or more interventions, potentially given at separate time points. Integrated surveys have the potential to conserve resources, minimize community interruption, and maximize the information gained. Whether for a single or integrated coverage survey, a best practice is to implement the CES as soon after the MDA as possible. Indeed, Budge et al.^6^ found that participant recall for triple drug MDA (albendazole, ivermectin, and praziquantel) was most accurate 1 month after MDA, but still relatively high 12 months post-MDA. When asking respondents to recall their participation in two or more campaigns, it is important that the survey teams are well trained in tactics that can help jog participants’ memories, such as showing examples of the different drugs, bringing a dose pole, and describing what the campaign team would have been wearing. The results presented here from Uganda suggest that participants differentially recalled their ingestion of albendazole and ivermectin, compared with azithromycin and praziquantel, which were given during two separate MDAs. These differential recall results were replicated across all three survey designs; because the three surveys took place in the same Ugandan district, this is good evidence of internal validity.

In 2016, on viewing the preliminary results from this study, the WHO Strategic and Technical Advisory Group for NTDs recommended that national NTD programs “implement CESs using PSS.”^35^ Since the release of this recommendation, several tools have been created to assist NTD programs in the design, implementation, and analysis of CES using PSS. An Excel-based CES Sample Builder tool helps programs determine the appropriate sample size, select the PSU, and identify the number of segments required in each PSU; a manual and training materials describing how to conduct CESs have been created; and to assist in the analysis and interpretation of the CES results, a Web-based Coverage Survey Analysis tool was developed.^36,37^ More recently, a job aid was created by a team of experts, in consultation with the WHO, to help guide NTD programs in deciding when, where, and why to use CESs in the context of ongoing MDAs.^38^

Coverage evaluation surveys are an essential tool in the monitoring and evaluation framework of NTD programs. The WHO has helped remove a barrier to the uptake of unbiased, statistically valid coverage evaluations by recommending a single, standardized method and providing resources and assistance to implementers. This study demonstrates that the PSS approach is feasible to implement, can be applied in a variety of country settings where the target population is spread out throughout the survey area, and a sampling frame exists with geographic units that can be used as PSUs and are accompanied by at least approximate estimates of total population or total households as a measure of size. Furthermore, the experience from Uganda and Honduras suggests that CESs can be used to conduct integrated monitoring across NTDs and other public health programs. By adopting the same sampling methodology as the immunization program, one can expect the familiarity with the PSS method and the number of survey teams trained in it to increase over time. The year 2020 is a pivotal year, with many program milestones being reevaluated and an increasing number of NTD programs performing at scale, CESs will remain an indispensable tool for monitoring the progress toward control and elimination goals and improving program effectiveness.

## Supplemental table

Supplemental materials

## References

[1] World Health Organization, 2012 Accelerating Work to Overcome the Global Impact of Neglected Tropical Diseases: A Roadmap for Implementation. Geneva, Switzerland: WHO, 1–42.

[2] Turner HC Walker M Churcher TS Osei-Atweneboana MY Biritwum N-K Hopkins A Prichard RK Basanez M-G, 2014 Reaching the London declaration on neglected tropical diseases goals for onchocerciasis: an economic evaluation of increasing the frequency of ivermectin treatment in Africa. Clin Infect Dis 59: 923–932. 24944228 10.1093/cid/ciu467 PMC4166981

[3] Winnen M Plaisier AP Alley ES Nagelkerke NJD van Oortmarssen G Boatin BA Habbema JDF, 2002 Can ivermectin mass treatments eliminate onchocerciasis in Africa? Bull World Health Organ 80: 384–391. 12077614 PMC2567795

[4] Irvine MA Reimer LJ Njenga SM Gunawardena S Kelly-Hope L Bockarie M Hollingsworth TD, 2015 Modelling strategies to break transmission of lymphatic filariasis–aggregation, adherence and vector competence greatly alter elimination. Parasit Vectors 8: 547. 26489753 10.1186/s13071-015-1152-3 PMC4618540

[5] Murray CJL Shengelia B Gupta N Moussavi S Tandon A Thieren M, 2003 Validity of reported vaccination coverage in 45 countries. Lancet 362: 1022–1027. 14522532 10.1016/S0140-6736(03)14411-X

[6] Budge PJ Sognikin E Akosa A Mathieu EM Deming M, 2016 Accuracy of coverage survey recall following an integrated mass drug administration for lymphatic filariasis, schistosomiasis, and soil-transmitted helminthiasis. PLoS Negl Trop Dis 10: e0004358. 26766287 10.1371/journal.pntd.0004358 PMC4713198

[7] World Health Organization, 2011 Monitoring and Epidemiological Assessment of Mass Drug Administration in the Global Programme to Eliminate Lymphatic Filariasis: A Manual for National Elimination Programmes. WHO/HTM/NT Available at: http://www.who.int/lymphatic_filariasis/resources/9789241501484/en/. Accessed June 7, 2018.

[8] World Health Organization, 2011 Helminth Control in School-Age Children. Geneva, Switzerland: WHO.

[9] World Health Organization, 1991 Training for Mid-level Managers: The EPI Coverage Survey. WHO/IVB/08 (WHO/EPI/MLM/91.10) Geneva, Switzerland: WHO.

[10] Brogan D Flagg EW Deming M Waldman R, 1994 Increasing the accuracy of the Expanded Programme on Immunization’s cluster survey design. Ann Epidemiol 4: 302–311. 7921320 10.1016/1047-2797(94)90086-8

[11] Valadez JJ Berendes S Lako R Gould S Vargas W Milner S, 2015 Finding the gap: revealing local disparities in coverage of maternal, newborn and child health services in South Sudan using lot quality assurance sampling. Trop Med Int Health 20: 1711–1721. 26432978 10.1111/tmi.12613

[12] Valadez JJ, 1991 Assessing Child Survival Programs in Developing Countries: Testing Lot Quality Assurance Sampling. Cambridge, United Kingdom: Harvard University Press.

[13] Harding E Beckworth C Fesselet J-F Lenglet A Lako R Valadez JJ, 2017 Using lot quality assurance sampling to assess access to water, sanitation and hygiene services in a refugee camp setting in South Sudan: a feasibility study. BMC Public Health 17: 643. 28789627 10.1186/s12889-017-4656-2 PMC5549393

[14] UNICEF, 2013 MICS5 Manual for Mapping and Household Listing. Available at: http://mics.unicef.org/tools#survey-design. Accessed June 8, 2018.

[15] ICF International, 2012 Demographic and Health Survey Sampling and Household Listing Manual. Calverton, MD: ICF International Available at: https://dhsprogram.com/pubs/pdf/DHSM4/DHS6_Sampling_Manual_Sept2012_DHSM. Accessed March 30, 2020.

[16] Dodge H Romig H, 1929 A method of sampling inspection. Bell Syst Tech J 8: 613–631.

[17] Robertson S Valadez JJ, 2006 Global review of health care surveys using lot quality assurance sampling (LQAS), 1984–2004. Soc Sci Med 63: 1648–1660. 16764978 10.1016/j.socscimed.2006.04.011

[18] UNICEF, 2000 Multiple Indicator Cluster Surveys. Available at: http://mics.unicef.org/tools. Accessed November 3, 2016.

[19] Turner AG Magnani RJ Shuaib M, 1996 A not quite as quick but much cleaner alternative to the Expanded Programme on Immunization (EPI) cluster survey design. Int J Epidemiol 25: 198–203. 8666490 10.1093/ije/25.1.198

[20] Pavluck A Chu B Mann Flueckiger R Ottesen E, 2014 Electronic data capture tools for global health programs: evolution of LINKS, an android-, web-based system. PLoS Negl Trop Dis 8: e2654. 24722343 10.1371/journal.pntd.0002654 PMC3983089

[21] Kish L, 1965 Survey Sampling. New York, NY: John Wiley & Sons Inc.

[22] Lumley T, 2010 Complex Surveys: A Guide to Analysis Using R. Hoboken, NJ: John Wiley and Sons.

[23] Lumley T, 2004 Analysis of complex survey samples. J Stat Softw 9: 1–19.

[24] Dietz V Venczel L Izurieta H Stroh G Zell ER Monterroso E Tambini G, 2004 Assessing and monitoring vaccination coverage levels: lessons from the Americas. Rev Panam Salud Publica 16: 432–442. 15673487 10.1590/s1020-49892004001200013

[25] Milligan P Njie A Bennett S, 2004 Comparison of two cluster sampling methods for health surveys in developing countries. Int J Epidemiol 33: 469–476. 15020569 10.1093/ije/dyh096

[26] Luman ET Worku A Berhane Y Martin R Cairns L, 2007 Comparison of two survey methodologies to assess vaccination coverage. Int J Epidemiol 36: 633–641. 17420165 10.1093/ije/dym025

[27] Hoshaw-Woodard S, 2001 Description and Comparison of the Methods of Cluster Sampling and Lot Quality Assurance Sampling to Assess Immunization Coverage. Geneva, Switzerland: WHO.

[28] Gong W Taighoon Shah M Firdous S Jarrett BA Moulton LH Moss WJ Hayford K O’Brien KL Chandir S, 2018 Comparison of three rapid household survey sampling methods for vaccination coverage assessment in a peri-urban setting in Pakistan. Int J Epidemiol 48: 583–595. 10.1093/ije/dyy263 30508112

[29] Baker MC 2010 Mapping, monitoring, and surveillance of neglected tropical diseases: towards a policy framework. Lancet 375: 231–238. 20109924 10.1016/S0140-6736(09)61458-6

[30] Cromwell EA Ngondi J McFarland D King JD Emerson PM, 2012 Methods for estimating population coverage of mass distribution programmes: a review of practices in relation to trachoma control. Trans R Soc Trop Med Hyg 106: 588–595. 22884927 10.1016/j.trstmh.2012.07.011

[31] Woodhall DM Mkwanda S Dembele M Lwanga H Drexler N Dubray C Harris J Worrell C Mathieu E, 2014 Exploring innovative ways to conduct coverage surveys for neglected tropical diseases in Malawi, Mali, and Uganda. Acta Trop 132: 119–124. 24462795 10.1016/j.actatropica.2014.01.001

[32] World Health Organization, 2018 Vaccination Coverage Cluster Surveys: Reference Manual. WHO/IVB/18.09 Geneva, Switzerland: WHO.

[33] Hund L Bedrick EJ Pagano M, Choosing a cluster sampling design for lot quality assurance sampling surveys. PLoS One 10: e0129564. 10.1371/journal.pone.0129564 PMC4488393 26125967

[34] Modi RN King C Bar-Zeev N Colbourn T, 2018 Caregiver recall in childhood vaccination surveys: systematic review of recall quality and use in low- and middle-income settings. Vaccine 36: 4161–4170. 29885771 10.1016/j.vaccine.2018.05.089

[35] World Health Organization, 2016 Report of the WHO Strategic and Technical Advisory Group for Neglected Tropical Diseases, April 12–13, 2016 Available at: http://www.who.int/neglected_diseases/NTD_STAG_report_2016.pdf?ua=1. Accessed June 7, 2018.

[36] Neglected Tropical Disease Support Center, 2018 Coverage Evaluation Surveys. Available at: https://www.ntdsupport.org/resources/coverage-survey-builder-coverage-evaluations. Accessed December 28, 2018.

[37] Secure Data Kit, 2018 Coverage Survey Analysis Tool. Available at: https://coverage.securedatakit.com/. Accessed December 28, 2018.

[38] NTD Toolbox, When to Use Which Key PC NTD M&E Tool? (Draft for Field-Testing). Available at: https://www.ntdtoolbox.org/toolbox-search/when-use-which-key-pc-ntd-me-tool-draft-field-testing. Accessed March 24, 2020.

