# Supplementary material for: A Multicountry Comparison of Three Coverage Evaluation Survey Sampling Methodologies for Neglected Tropical Diseases
Source: Am J Trop Med Hyg. 2020 Aug 24;103(4):1700–10. doi: 10.4269/ajtmh.19-0946 (PMC7543869; doi:10.4269/ajtmh.19-0946)
Supplement: Supplementary file 1 [file tpmd190946.SD1.pdf]

Supplemental Table 1. WHO target coverage threshold by disease along with the LQAS decision rule to classify each supervisory area as above/below the WHO target threshold and the associated Type 1 error.

**Disease**	**WHO minimum target coverage threshold**	**Denominator for WHO target coverage**	**Number of people who reported swallowing the pills (out of 19)**	**Confidence level (Type 1 error)**
Lymphatic filariasis	65%	Entire population	≥15	94%
Onchocerciasis	65%[a]	Entire population	≥15	94%
Schistosomiasis	75%	School age children	≥17	97%
Soil Transmitted Helminths	75%	School age children	≥17	97%
Trachoma	80%	Entire population	≥17	92%

WHO = World Health Organization

[a]Note that treating 65% of the entire population in the endemic area is the minimum target coverage threshold (referred to as ‘epidemiologic coverage’); some WHO documents reference an 80% target threshold for onchocerciasis , which corresponds when the goal is elimination of ocular morbidity (reference: https://www.who.int/neglected_diseases/resources/9789241516464/en/)
